# The impacts of college educational satisfaction and helpfulness of career support on life satisfaction among Korean youth: The mediating role of mental health

**DOI:** 10.1371/journal.pone.0296702

**Published:** 2024-01-05

**Authors:** Sangmi Lee

**Affiliations:** Department of Nursing, College of Nursing, Dongyang University, Yeongju-si, Gyeongsangbuk-do, Republic of Korea; National Taiwan University of Arts, TAIWAN

## Abstract

This study aimed to investigate the structural relationships between college educational satisfaction, the helpfulness of career support, and mental health, and how these factors influence the life satisfaction of late adolescents and young adults. The study utilized data from 550 Korean individuals 18–24 years of age who have experienced going to college, collected in the “2021 Youth Socio-Economic Reality Survey” conducted by the National Youth Policy Institute. Data analysis was conducted with SmartPLS 3.0 software, using a structural equation model with the partial least squares method. The mediating impact of mental health was validated using bootstrapping. The study yielded several key findings. First, college educational satisfaction, the helpfulness of career support, and mental health all exerted a significant and positive influence on the life satisfaction of young people. Second, college educational satisfaction was found to significantly positively affect youth’s mental health. Third, mental health was identified as playing a significant positive mediating role in the connection between college educational satisfaction and the life satisfaction of young people. The study underscores the importance of enhancing mental health, alongside improving college educational satisfaction and career support, to boost the life satisfaction of young people. Suggestions based on these findings are discussed.

## Introduction

As societies become more economically advanced and modernized, the stages between childhood and adulthood have become increasingly distinct [1]. This has led to a growing need to understand late adolescence and young adulthood. The period between the ages of 18 and 24, known as late adolescence and young adulthood, is a critical time when brain development is completed and individuals assume greater social responsibilities as they transition from childhood to adulthood [2]. Moreover, most mental health issues manifest before the age of 24, and health and well-being problems that arise during late adolescence and young adulthood, such as the onset of health risk behaviors like smoking and poor dietary habits leading to chronic diseases, can persist into adulthood [3, 4]. Therefore, it is crucial to investigate the factors influencing life satisfaction during this period. Life satisfaction pertains to a person’s subjective evaluation of their objective life circumstances [5]. It has been reported that life satisfaction declines most rapidly during late adolescence and young adulthood, compared to other stages of human life [6]. Therefore, although the management of the life satisfaction of young people should be emphasized, research on this topic is limited.

Life satisfaction among Koreans has been reported to be very low. In 2023, the mean life satisfaction score for Koreans was found to be 5.8 points. This score was not only lower than the Organization for Economic Co-operation and Development (OECD) average of 6.7 points, but it also ranked as almost the lowest among the 38 OECD countries, placing 36th [5]. The happiness of Korean youth was also found to be the lowest among the 11 countries surveyed by the OECD [7]. Therefore, it is crucial to identify the factors contributing to this situation. Korea, which has experienced faster economic growth over the past 50 years than any other country, boasts the highest college entrance rate among OECD countries. This surge in educational attainment among young people has led to a mismatch in the labor market due to a shortage of jobs that align with their qualifications [8]. This issue, coupled with the rise in the economically inactive population among Korean youth, is placing an excessive burden on employment [8]. Furthermore, the primary cause of burnout among young people is reported to be anxiety about their future careers, accounting for 53.3% [9].

Anxiety about an uncertain future, particularly in relation to the career paths of young people, is a significant factor that can decrease life satisfaction [5, 10]. Previous studies have shown that in adolescents, higher career anxiety [10], career stress [11], and career decision-making difficulties [12] tend to be associated with lower life satisfaction. Conversely, an increased sense of efficacy [11] and career identity [13] can significantly boost life satisfaction. This substantiates the pivotal role of career-related factors in the life satisfaction of young people. In response to this, schools and communities in Korea have recently begun offering education, counseling, and experiential programs to support the career paths of young people. However, there is a scarcity of research investigating the influence of these career support programs on youth life satisfaction [14, 15]. As such, it is important to evaluate the direct effects of the current career support programs in Korea on the life satisfaction of late adolescents, in order to provide informed suggestions for the direction of future career support programs.

Educational satisfaction refers to the extent to which students are content with their school education in general or the practical benefits of education for their current and future lives [16]. The study’s target population, late adolescents and young adults, are preparing for full-scale socio-economic activities through college education. Therefore, educational experiences during this period are a significant part of their lives, constituting a large portion of their life experiences. The university enrollment in Korea is approximately 75%, meaning that the majority of young people experience college education [17]. Thus, college education plays a significant role in the lives of young people in Korea. One study in Korea found that satisfaction with college education had a long-term impact on the life satisfaction of college graduates [14]. However, there are very few studies examining the impact of college educational satisfaction on life satisfaction.

Mental health refers to a state of well-being that extends beyond the mere absence of mental disorders or diseases. It enables an individual to utilize their abilities, manage daily life stress, engage in productive work, and contribute to their community [18]. Late adolescents and young adults are particularly susceptible to mental health issues as their brains are still developing. The incidence of mental health issues, including anxiety and depression, notably increases in late adolescence, peaking between the ages of 20 and 24 [19]. However, the World Health Organization reports that 10% of children and adolescents experience mental health problems, and the majority of them do not receive appropriate diagnosis and treatment [20]. In Korea, the suicide rate is the highest among OECD countries, with a rapid increase observed among individuals in their late teens and twenties [5]. This trend is believed to be linked to mental health issues, given the rise in perceived stress and experiences of depression during this period [18]. Mental health problems pose serious threats, disrupting the regular life patterns of teenagers and diminishing their quality of life, and mental health has been extensively studied as an important factor related to the life satisfaction of youth [11, 13].

Career-related factors and educational experiences during late adolescence and young adulthood have a significant correlation with mental health. Negative perceptions about one’s career, such as a high degree of uncertainty [21] or experiencing excessive work-related stress [22], can negatively impact the mental health of young individuals. Specifically, reports indicate that young people in Korea often experience negative emotions such as anxiety, helplessness, depression, and frustration during their job search activities, as per the results of the 2022 Youth Job Perception Survey. Korea has recently been focusing on providing support for career preparation, including career education, planning, and experience, primarily from elementary school through college, with efforts underway to enhance these initiatives [23]. Therefore, it is now crucial to assess the practical effectiveness of these career support programs for youth in terms of promoting mental health. In addition, high academic achievement in adolescence has been shown to reduce depression and anxiety, while also increasing emotional regulation [24]. However, high levels of examination stress can lead to an increase in mental health problems and diminished psychological well-being among adolescents [25, 26]. Previous research suggests that satisfaction with education during youth may be closely related to mental health, although this relationship has not been extensively studied to our knowledge.

Life satisfaction is a complex concept, influenced and shaped by various internal and external individual characteristics [27]. The developmental period of late adolescence and young adulthood is a preparatory stage just before one enters society in earnest, and it is particularly important to investigate the impact of significant external features, such as college education and career support experiences, on life satisfaction in this stage of life. An understanding of these issues is crucial for devising strategies to improve environmental factors with a positive impact on life satisfaction. However, little research has been conducted on this topic. Furthermore, it is highly meaningful to examine the structural relationships that underpin how mental health, a significant internal characteristic known to be highly vulnerable and influential on life satisfaction in late adolescents and young people, mediates the relationship between variables of college education satisfaction, helpfulness of career support, and life satisfaction. Exploring the role of mental health as a mediator can be considered a crucial step in elucidating the mechanisms between internal and external factors influencing youth’s life satisfaction. As far as the researcher is aware, this remains an area that has not yet been extensively studied and is worth exploring.

### Study purpose

The current study aimed to explore the structural relationships among factors influencing life satisfaction in late adolescents and young adults. The specific research objectives, targeted at adolescents and young adults, are as follows. First, we aimed to determine the impact of education satisfaction and the helpfulness of career support on life satisfaction. Second, we sought to understand the effect of education satisfaction and the helpfulness of career support on mental health. Third, we aimed to determine the impact of mental health on life satisfaction. Fourth, we sought to identify the mediating effect of mental health in the association between education satisfaction, the helpfulness of career support, and life satisfaction.

## Prior research and research hypotheses

### Educational satisfaction → mental health and life satisfaction

School is the environment that is closest to students and significantly influences their health and development [28]. This is particularly true for mental health. Connections with teachers [29] and contentment with school life [25] have been identified as factors significantly contributing to adolescents’ mental health in a previous study. Conversely, the stress associated with exams has been shown to negatively affect mental health [25]. Furthermore, those with low academic achievement are at an elevated risk of encountering mental health issues, as evidenced by increased daily stress, lower sleep satisfaction, depression, and suicidal thoughts [30]. Therefore, a positive correlation is anticipated to exist between adolescents’ satisfaction with their education and their mental health. However, research in this specific area remains scarce.

A previous study on the association between satisfaction with education and life satisfaction found that satisfaction with college education exhibited a notable positive influence on the life satisfaction of present employees after graduating from college [14]. Furthermore, the concept of school adaptation, which is akin to educational satisfaction, was identified as a factor predicted life satisfaction among adolescents on the verge of graduating from middle and high school [31]. This factor also exerted a positive influence on the life satisfaction of middle school students [13]. Moreover, a positive correlation was identified between academic achievement and school adaptation in transitional adolescents and their life satisfaction [32]. Drawing from these previous studies, the following hypotheses have been formulated for this study.

Hypothesis 1 (H1). College educational satisfaction will significantly and positively influence youth’s mental health.

Hypothesis 2 (H2). College educational satisfaction will significantly and positively influence youth’s life satisfaction.

### Helpfulness of career support → mental health, life satisfaction

Previous research exploring the association between career-related factors and mental health revealed that awareness of career barriers positively influenced mental health problems in female college students [21]. Furthermore, employment and career stress were identified as significant factors that heightened depression in college students [22]. The career identity of college students significantly and positively influenced their ability to tolerate uncertainty [33]. Similarly, research with Turkish university students [34] and European middle school students [35] found that career adaptability positively influenced resilience. Additionally, significant changes were observed in the happiness scores of adolescents before and after their participation in a career exploration program [15]. These studies underscore the significant relationship between career-related factors and mental health.

Career planning is a crucial task during adolescence, as it allows individuals to shape their life’s direction based on self-understanding [13, 31]. This process can reflect one’s perception of, and relationship with, their life. Prior research has indicated that the career identity of adolescents nearing graduation from middle and high school can significantly predict life satisfaction [31]. It has been observed to exert a significant positive influence on the life satisfaction of middle school students [13]. Furthermore, factors such as self-efficacy in career decision-making, career satisfaction, career preparation behavior, and career indecision have been identified as significant predictors of college students’ life satisfaction [3]. In a study encompassing middle school, high school, and college students, career decision difficulty was found to negatively impact life satisfaction [12]. Moreover, it has been reported that the satisfaction of out-of-school youth with the use of support centers significantly enhances life satisfaction through the development of career capabilities [36]. Building upon the findings of prior research, the following hypotheses were developed for the current study.

Hypothesis 3 (H3). The helpfulness of career support perceived by youth will significantly and positively influence mental health.

Hypothesis 4 (H4). The perceived helpfulness of career support by youth will significantly and positively influence life satisfaction.

### Mental health → life satisfaction

Research into adolescents’ mental health has explored the roles of emotions, stress, and depression, revealing a significant correlation with life satisfaction. It has been found that positive emotions in college students [37] and high school students [11] significantly enhance life satisfaction. Moreover, effective emotion regulation has been shown to positively impact life satisfaction [38], whereas emotional issues significantly detract from the life satisfaction of middle school students [13]. Daily stressors, including those related to parents, school, friends, appearance, and personal circumstances, have been found to negatively affect life satisfaction [38, 39]. Emotional problems, such as issues with attention, aggression, physical symptoms, and social withdrawal, also significantly undermine the life satisfaction of college students [40]. Depression, a prevalent mental health issue, has been shown to significantly reduce adolescents’ life satisfaction [41]. Notably, in groups where depression escalated from middle school through high school, life satisfaction was found to be lower upon reaching college [42]. Mental health factors, such as loneliness and depression during the COVID-19 era, significantly negatively impacted the life satisfaction of young people in South Africa [43]. Based on these findings, the following hypothesis for this study was established.

Hypothesis 5 (H5). Mental health will significantly and positively influence youth’s life satisfaction.

### The mediating role of mental health

There is scarce research on the mediating role of mental health in the associations of education satisfaction and career support-related factors with life satisfaction among young people. Previous research has shown that mental health functions as a significant mediator in the relationship between life satisfaction and influencing factors among young people. A prior study revealed a noteworthy mediating influence of emotion regulation on the connection between stress and adolescents’ life satisfaction [38]. Furthermore, it was found that positive emotions in Korean college students significantly mediated the association of social support with life satisfaction [37]. Similarly, a study conducted with Chinese youth in Malaysia revealed that loneliness had a partial mediating effect in the association of perceived social support with life satisfaction [44]. Furthermore, resilience, which is indicative of mental strength, was found to partially mediate the relationship between perceived emotional intelligence and life satisfaction in adolescents [45]. In the context of the relationship between loneliness during the COVID-19 era and life satisfaction among young people in South Africa, depression was found to have a partial mediating effect [43]. Additionally, in a study involving workers in the United States, poor mental health fully mediated the impact of organizational and supervisor support on life satisfaction [34].

Prior studies have explored the mediating effects of mental health factors in the relationship between career-related variables and life satisfaction. For instance, a study focused on Korean college students found significant mediating effects of tolerance for uncertainty and positive/negative affect in the impact of career identity on life satisfaction [33]. Additionally, in a study exploring the impact of career adaptability on life satisfaction among Turkish university students, resilience played a partial mediating role [46]. Similarly, in a study involving European middle school students, resilience showed a significant mediating effect in the association between career adaptability and life satisfaction [35]. Based on these findings, the following hypothesis for this study was established.

Hypothesis 6 (H6). The satisfaction with college education of youth will significantly and positively affect life satisfaction, with mental health serving as a mediating factor.

Hypothesis 7 (H7). The perceived helpfulness of career support by youth will significantly and positively affect life satisfaction, with mental health serving as a mediating factor.

**Fig 1:**
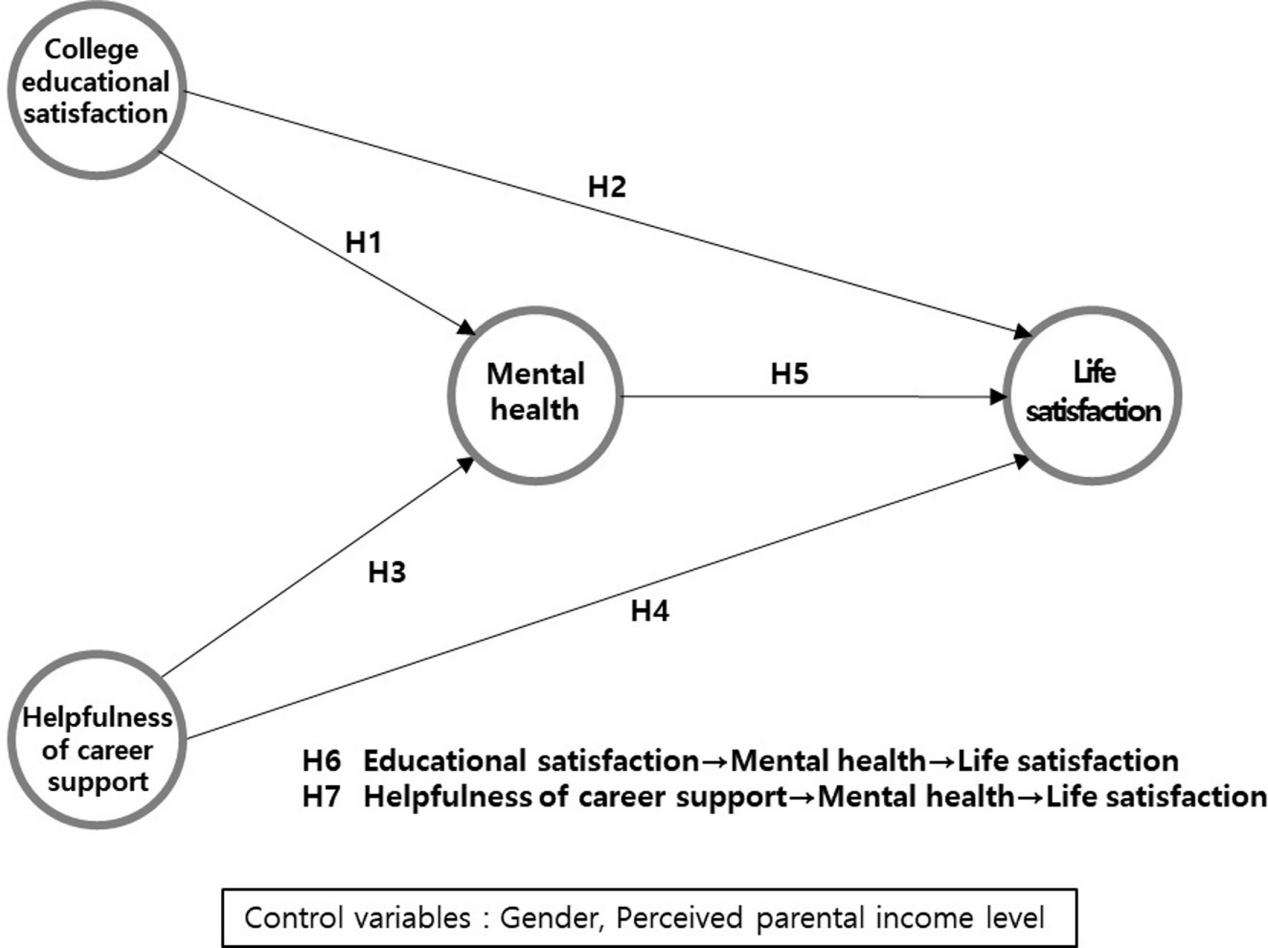
Research model and hypothesis.

Based on the previous studies reviewed, the research model and hypothesis of this study are depicted in Fig 1.

## Methods

### Study design

The current study was designed as secondary analysis research using data from the “2021 Youth Socio-Economic Reality Survey” to explore the structural association among satisfaction with college education, the helpfulness of career support, and mental health, all of which impact the life satisfaction of young people.

### Subjects and data collection

In this study, using data from the "2021 Youth Socio-Economic Reality Survey" provided by the National Youth Policy Institute, we examined the factors that influenced life satisfaction in late adolescents and young adults [16]. The survey extracted 290 sample districts using a stratified sampling method, with the survey districts of the 2019 Statistics Korea Population and Housing Census serving as the sampling frame. Households and youth household members were then selected from each survey district according to the proportion of sample districts in each stratum. The survey was conducted with 2,041 young individuals aged 15 to 39. However, this study specifically used data from 550 individuals who have experienced college education among 773 individuals aged 18 to 24, representing late adolescents and young adults. Data collection took place from June to August 2021, with face-to-face individual interviews conducted using a tablet PC during a researcher’s household visit.

### Ethical considerations

This study was processed after ethical approval by the Institutional Review Board (IRB No.:1041495-202308-HR-01-01) of the researcher’s affiliated institution. This exemption from ethics review was granted because the study is a secondary analysis of publicly available, non-identifiable pre-existing data. The data were sourced from the 2021 Youth Socio-Economic Reality Survey conducted by the NYPI in Korea (https://www.nypi.re.kr/archive/board?menuId=MENU00472).

### Measurements

This study’s research tools used questions developed for the “2021 Youth Socio-Economic Reality Survey” according to the definitions of the survey [16].

#### College educational satisfaction

College educational satisfaction was assessed using four questions: overall satisfaction with college education, its helpfulness in future life, its helpfulness in securing employment, and its relevance to the chosen major (field). Each question was evaluated by a 5-point Likert scale, with 1 denoting “not at all” and 5 denoting “very much so.” A higher score represented a greater level of satisfaction with college education. The reliability coefficient (Cronbach’s α) for the tool utilized in this study was recorded at 0.82.

#### Helpfulness of career support

The helpfulness of career support was assessed using six questions that gauged the perceived usefulness of provided programs related to career, job, and employment (such as counseling, classes, experiences, etc.). There were three items for each two sub-domains (one domain including experience and classes related to career or vocation, and linkage with employment agencies and the other domain including counseling and briefing sessions or career/job fairs, as well as major experience). Each question was evaluated using a 5-point Likert scale, with 1 denoting “not at all” and 5 denoting “very much so.” A higher score signifies a higher level of perceived career support. The reliability coefficient (Cronbach’s α) for the tool utilized in this study was recorded at 0.77.

#### Mental health

Mental health was assessed using five questions related to participants’ daily mood, vitality, comfort, and interest, exemplified by statements such as “I feel bright and happy.” Each question was evaluated using a 6-point Likert scale, spanning from 0 ("not at all") to 5 ("always"), with a higher score indicating a higher level of subjective mental health. The reliability coefficient (Cronbach’s α) for the tool utilized in this study was recorded at 0.90.

#### Life satisfaction

Life satisfaction pertains to the extent of contentment with various aspects of one’s life, including your standard of living, health, personal achievements, familial and friendly relationships, overall interpersonal interactions, safety, future stability, leisure time, neighborhood environment, and your overall life experience. This concept was evaluated using 11 distinct questions. For each of the three sub-domains (overall life or interpersonal relationships or time to spare, achievement or future stability, and health or safety), there were three to four items. Each question was rated on an 11-point scale, where 0 signifies no satisfaction at all and 10 represents the highest level of satisfaction. Therefore, higher scores denote greater life satisfaction. The reliability coefficient (Cronbach’s α) for the tool utilized in this study was recorded at 0.91.

#### Control variables

Significant differences were observed in adolescents’ mental health [25] and life satisfaction [40] based on their gender. Additionally, their income or economic level significantly influenced their life satisfaction [5, 24], depression, anxiety, and emotional regulation [24]. Previous research findings, which highlighted these significant differences, led to the selection of gender and parents’ income level as control variables in this study. Consequently, gender was coded as a dummy variable, with “male” assigned “0” and “female” assigned “1.” Parental income level was gauged on a 10-point scale, where 1 point represented the lowest perceived income level, and 10 points represented the highest. A higher score denoted a greater level of parental income.

### Data analysis

In this study, data were processed using SPSS 26.0 (IBM Corp., Armonk, NY, USA) and SmartPLS 3.0 (SmartPLS GmbH, Germany). Basic statistics, including mean, standard deviation, frequency, percentage, skewness, and kurtosis, were employed to analyze the general characteristics of the study subjects and main variables. Pearson correlation coefficients were utilized to examine correlations between major variables. A structural equation model based on the partial least squares (PLS-SEM) technique was employed to analyze the relationships regarding influence between major variables, and the mediating effect was assessed through bootstrapping.

To assess the overall measurement model using PLS-SEM, we evaluated convergent validity, discriminant validity, and internal consistency reliability. In addition, to evaluate the structural model, we calculated χ^2^ (chi-square) and the standardized root mean square residual (SRMR). Furthermore, we assessed the R^2^, f^2^, and Q^2^ as part of the evaluation of the structural model. In order to validate the hypotheses of the structural model, we computed the path coefficient, standard deviation, t-value, and p-value for each hypothesized relationship between variables.

## Results

### General characteristics of the participants

Among all participants (550) in this study, the proportion of male and female subjects was equal, at 50% each. The mean age was 21.43 years (±1.84), and a significant majority, 380 individuals or 70.2%, were attending college. The subjects’ perceived average level of parental income was 6.08 points (±1.38) (Table 1).

**Table 1:** Baseline characteristics of participants (N = 550).

Characteristics		n (%)	Mean±SD	Range
**Gender**	**Male**	275 (50.0)	-	-
	**Female**	275 (50.0)	-	-
**College attendance status**	**Graduation**	143 (26.0)	-	-
	**Attending (including a leave of absence)**	386 (70.2)		
	**Dropout**	21 (3.8)	-	-
**Age (years)**	**-**	-	21.43±1.84	18–24
**Perceived parental income level**	**-**	-	6.08±1.38	1–10

### Correlation relationships and basic statistics of the major variables

The mean scores for the primary variables ranged from 3.44 points (±0.88) to 3.70 points (±0.82) for college educational satisfaction, and from 3.36 points (±0.76) to 3.47 points (±0.69) for the helpfulness of career support. The mean scores for mental health were between 2.69 points (±1.34) and 3.52 points (±1.13), while life satisfaction scored from 6.51 points (±1.29) to 7.21 points (±1.36). The absolute skewness of the main variables was between 0.28 and 1.17, which is less than the standard value of 2, indicating a normal distribution. Similarly, the absolute kurtosis ranged from 0.03 to 3.70, which is less than the standard value of 7, also meeting the criteria for normality [47]. Most of the primary variables demonstrated statistically significant positive correlations except for one relationship—namely, that between helpfulness of career support and mental health (Table 2).

**Table 2:** Correlation relationships and basic statistics of the major variables.

Correlation		College educational satisfaction				Helpfulness of career support		Mental health					Life satisfaction		
		➀	➁	➂	➃	➀	➁	➀	➁	➂	➃	➄	➀	➁	➂
**College educational satisfaction**	**➀**														
	**➁**	0.62^**^													
	**➂**	0.51^**^	0.56^**^												
	**➃**	0.52^**^	0.57^**^	0.46^**^											
**Helpfulness of career support**	**➀**	0.27^**^	0.27^**^	0.37^**^	0.24^**^										
	**➁**	0.30^**^	0.28^**^	0.35^**^	0.26^**^	0.71^**^									
**Mental health**	**➀**	0.23^**^	0.19^**^	0.16^**^	0.11^*^	0.12^*^	0.12^**^								
	**➁**	0.22^**^	0.17^**^	0.18^**^	0.13^**^	0.11	0.11^*^	0.69^**^							
	**➂**	0.20^**^	0.20^**^	0.20^**^	0.12^**^	0.16^**^	0.13^**^	0.83^**^	0.66^**^						
	**➃**	0.21^**^	0.20^**^	0.11^*^	0.10^*^	0.19^**^	0.20^**^	0.57^**^	0.59^**^	0.59^**^					
	**➄**	0.21^**^	0.21^**^	0.19^**^	0.16^**^	0.15^**^	0.19^**^	0.63^**^	0.54^**^	0.69^**^	0.58^**^				
**Life satisfaction**	**➀**	0.27^**^	0.22^**^	0.21^**^	0.23^**^	0.27^**^	0.26^**^	0.33^**^	0.33^**^	0.36^**^	0.25^**^	0.31^**^			
	**➁**	0.32^**^	0.25^**^	0.23^**^	0.25^**^	0.31^**^	0.25^**^	0.36^**^	0.38^**^	0.39^**^	0.34^**^	0.32^**^	0.82^**^		
	**➂**	0.27^**^	0.24^**^	0.26^**^	0.20^**^	0.27^**^	0.29^**^	0.34^**^	0.36^**^	0.37^**^	0.30^**^	0.25^**^	0.78^**^	0.72^**^	
**Mean**		3.44	3.66	3.70	3.58	3.36	3.47	3.49	3.52	3.32	3.00	2.69	6.51	6.52	7.21
**SD**		0.80	0.83	0.82	0.84	0.76	0.69	1.12	1.13	1.22	1.31	1.34	1.29	1.31	1.36
**Skewness**		-0.64	-0.66	-0.82	-0.48	-0.28	-0.75	-1.17	-0.89	-0.80	-0.72	-0.29	-0.70	-0.68	-1.17
**Kurtosis**		0.71	0.41	0.92	0.03	0.43	0.89	0.70	0.37	0.00	-0.29	-0.95	2.41	1.98	3.70

^*^p < .05

^**^p < .01 / ➀, ➁, ➂, ➃, ➄ (Observed variables for each latent variable)

### Evaluation of the measurement model fit

The fit of the measurement model used in this study was validated through convergent validity, internal consistency reliability, and discriminant validity. To evaluate the convergent validity of the measurement model, the outer loading relevance, with a reference value of >0.7, exhibited variation between 0.76 and 0.94 and the average variance extracted (AVE), with a reference value of >0.5, varied from 0.65 to 0.85. These results met the standard values, thereby confirming convergent validity [48, 49]. The internal consistency reliability was confirmed because Cronbach’s α (0.77 to 0.91), rho_A (0.77 to 0.92), and composite reliability (0.88 to 0.94) all met the standard reference value of >0.7 [48], as shown in Table 3. Furthermore, discriminant validity was confirmed because the Fornell-Larcker criteria were met, according to which the square roots of the AVE for the latent constructs were consistently greater than the correlation coefficients between the latent constructs [50] (Table 4).

**Table 3:** Convergent validity and internal consistency reliability in the measurement model.

Latent variables		Outer loading	Cronbach’s α	rho_A	Composite reliability	Average variance extracted
**College educational satisfaction**	**Overall satisfaction**	0.84	0.82	0.84	0.88	0.65
	**Helpfulness in future life**	0.85				
	**Helpfulness in securing employment**	0.78				
	**Relevance to the chosen major (field)**	0.76				
**Helpfulness of career support**	**Experience or classes related to career and vocation, and linkage with employment agencies**	0.89	0.77	0.77	0.90	0.81
	**Counseling and briefing sessions or career/job fairs, and major experience**	0.91				
**Mental health**	**Feeling bright and happy**	0.89	0.90	0.90	0.93	0.71
	**Feeling calm and comfortable**	0.83				
	**Feeling active and energetic**	0.90				
	**No fatigue and feeling refreshed after sleep**	0.78				
	**A daily life full of excitement**	0.81				
**Life satisfaction**	**Overall life or interpersonal relationships or time to spare**	0.94	0.91	0.92	0.94	0.85
	**Achievement or future stability**	0.93				
	**Health or safety**	0.90				

**Table 4:** Discriminant validity in the measurement model.

Latent variable	College educational satisfaction	Helpfulness of career support	Mental health	Life satisfaction
**College educational satisfaction**	0.81			
**Helpfulness of career support**	0.33	0.90		
**Mental health**	0.26	0.16	0.84	
**Life satisfaction**	0.33	0.29	0.43	0.92

### Evaluation of the structural model fit and hypothesis verification

Structural model fit was confirmed by a χ^2^ value 675.80 and an SRMR value of 0.048, meeting the reference value of <0.08. The variance inflation factor of the study’s structural model was 1.596–3.955, which is lower than the standard value of 5, indicating that multicollinearity among the latent variables was not a problem [48]. The adjusted R^2^ values for mental health and life satisfaction were 0.10 and 0.33, respectively. According to Cohen’s [51] suggestion for interpreting the R^2^ values of endogenous latent variables (weak = 0.02, moderate = 0.13, substantial = 0.26), the explanatory power of mental health was approximately moderate and explanatory power of life satisfaction was more than substantial. The effect sizes (f^2^) of college educational satisfaction and career support helpfulness on mental health were 0.039 and 0.006, respectively. The effect sizes (f^2^) of college educational satisfaction, career support helpfulness, and mental health on life satisfaction were 0.026, 0.026, and 0.135, respectively. The predictor variable’s influence is deemed high at the structural level when its f^2^ value is 0.35, is considered medium at 0.15, and is characterized as small at 0.02, according to Cohen [51]. Mental health had the largest effect size (f^2^) on life satisfaction, which can be interpreted as an approximately medium effect [43, 52]. The Q^2^ values, reflecting the predictive relevance in the structural model, were 0.066 for mental health and 0.266 for life satisfaction, both meeting the reference value of above 0 [52, 53]. This confirms the predictive suitability of the study’s structural model.

**Fig 2:**
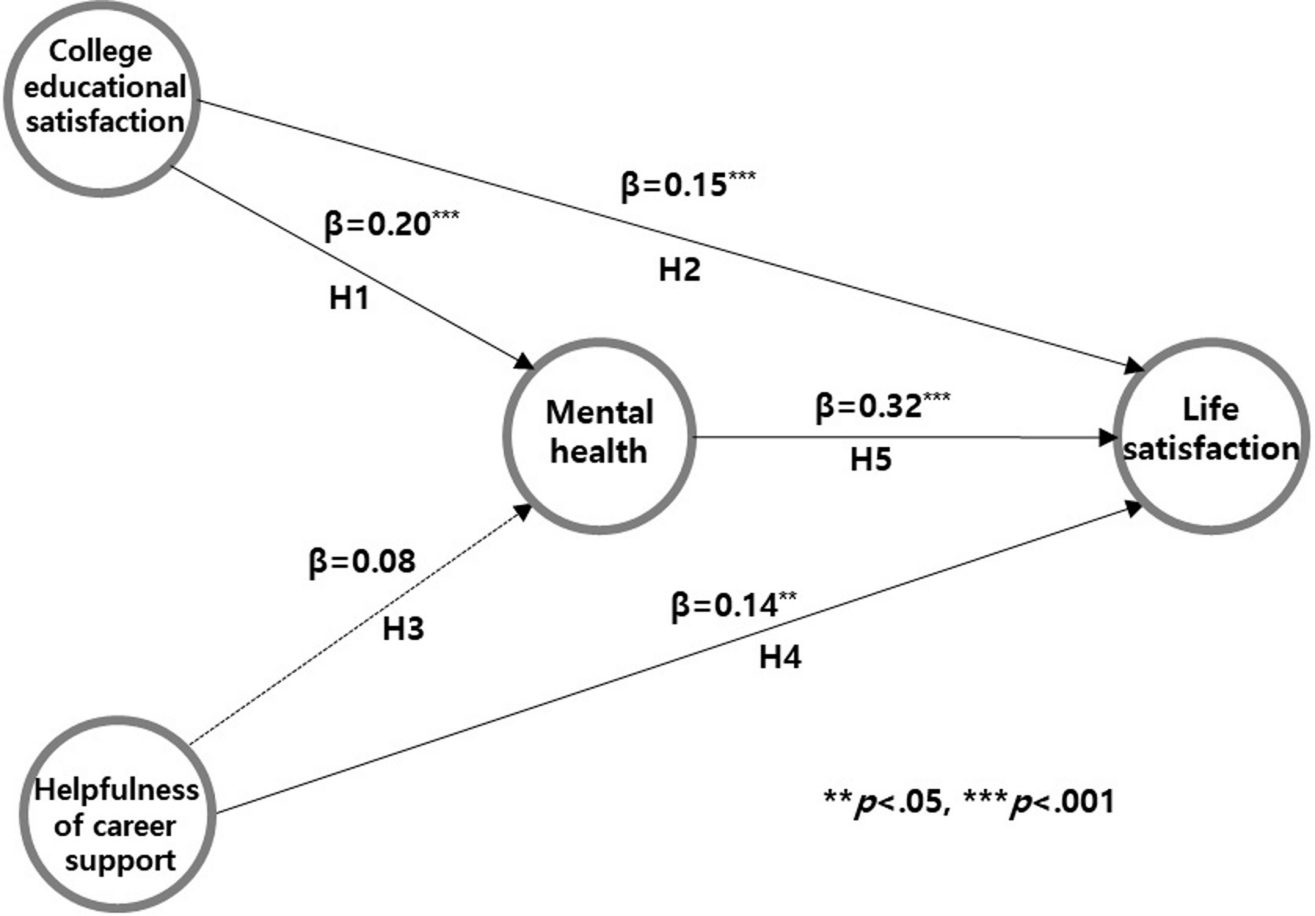
Results of research hypothesis testing for the structural model.

Upon testing the hypothesis regarding the direct effect of the structural model (Table 5) (Fig 2), it was found that college educational satisfaction significantly and positively influenced both mental health (β = 0.20, p < .001) and life satisfaction (β = 0.15, p < .001). Furthermore, the helpfulness of career significantly and positively influenced life satisfaction (β = 0.14, p = .001), but it did not significantly affect mental health. Mental health, however, did exhibit a significant positive impact on life satisfaction (β = 0.32, p < .001). Gender, which was a control variable, did not significantly influence either mental health or life satisfaction. Parental income level, in contrast, significantly and positively influenced both mental health (β = 0.14, p = .002) and life satisfaction (β = 0.26, p < .001).

**Table 5:** Research hypothesis testing.

Research hypothesis		Path coefficient	SD	t (*p*)	Judgment
**Direct effects**					
**H1**	**College educational satisfaction → Mental health**	0.20	0.05	3.97 (< .001)	Supported
**H2**	**College educational satisfaction → Life satisfaction**	0.15	0.04	3.40 (< .001)	Supported
**H3**	**Helpfulness of career support → Mental health**	0.08	0.06	1.32 (.186)	Not supported
**H4**	**Helpfulness of career support → Life satisfaction**	0.14	0.04	3.25 (.001)	Supported
**H5**	**Mental health → Life satisfaction**	0.32	0.04	8.24 (< .001)	Supported
**Control variables**	**Gender → Mental health**	-0.06	0.04	1.52 (.129)	Not supported
	**Gender → Life satisfaction**	0.02	0.03	0.58 (.559)	Not supported
	**Parental income level → Mental health**	0.14	0.04	3.11 (.002)	Supported
	**Parental income level → Life satisfaction**	0.26	0.04	7.11 (< .001)	Supported
**Indirect effects**					
**H6**	**Education satisfaction → Mental health → Life satisfaction**	0.06	0.02	3.55 (< .001)	Supported
**H7**	**Helpfulness of career support → Mental health → Life satisfaction**	0.02	0.02	1.29 (.197)	Not supported

Upon evaluating the hypothesis concerning the mediating impact within the structural model (Table 5), it was found that college educational satisfaction significantly and positively influenced life satisfaction, with mental health acting as a mediator (β = 0.06, p < .001). However, the impact of career support’s helpfulness on life satisfaction, mediated by mental health, was not statistically significant.

## Discussion

This study, which utilized gender and parental income level as control variables, aimed to explore the structural association between college educational satisfaction, the perceived helpfulness of career support, and mental health, all of which influence life satisfaction in late adolescents and young adults. The findings and recommendations from this study are presented below.

First, in this study, satisfaction with college education significantly and positively impacted life satisfaction in late adolescents and young adults (β = 0.15, p < .001). This result is consistent with prior research, according to which factors such as school adaptation and academic achievement, which are closely related to educational satisfaction, similarly exhibited a significant positive influence on life satisfaction [13, 17, 31]. In addition, this parallels a previous finding [14] that satisfaction with college education significantly and positively affects the life satisfaction of college graduates. Thus, this report provides evidence that satisfaction with one’s college education plays a paramount role in determining life satisfaction during late adolescence and young adulthood.

Second, the level of career support provided to young people significantly and positively impacted their life satisfaction (β = 0.14, p = .001). This finding contradicts the results of a previous study [14], which found no significant correlation between participation in career and employment programs and the life satisfaction of employee post-college graduation. However, our results align with another previous study [15], which found that participation in career exploration programs effectively improved adolescents’ happiness. Therefore, subsequent studies should validate the relationship between the level of career support and life satisfaction. Moreover, our study suggests that it is the positive perception of the degree of helpfulness of career support, rather than participation in the career support program itself, that is related to life satisfaction. Our findings also suggest the importance of increasing satisfaction by delivering a career support program tailored to students’ specific needs, aptitudes, and majors, thereby providing practical assistance.

Third, this study found that satisfaction with college education significantly and positively influenced the mental health of young people (β = 0.20, p < .001). This corresponds to the outcomes of Lee et al. [25], indicating that school satisfaction, a concept similar to educational satisfaction, significantly and positively influences young people’s mental health. Studies exploring the relationship between college educational satisfaction and mental health are scarce, making this study a valuable confirmation of the importance of strategies that encourage young people to view their college education positively and engage actively in the educational process for the sake of their mental health. Furthermore, the outcomes of this study indicate that when adolescents perceive education as a crucial and advantageous process for their future, their mental health tends to improve. Therefore, when evaluating the mental health of adolescents, it is also important to evaluate their level of satisfaction with the school curriculum and school life.

Fourth, we found that mental health not only significantly positively influenced the life satisfaction of late adolescents and young adults, but it also appeared to have the most substantial impact (β = 0.32, p < .001). This aligns with previous research findings that positive emotions significantly enhance life satisfaction among college [37] and high school students [11], and that mental health issues like depression and stress significantly detract from life satisfaction. Furthermore, our study identified youth’s mental health as a mediator of the relationship between college educational satisfaction and life satisfaction. This implies that satisfaction with college education positively influences mental health, subsequently contributing to an increase in life satisfaction. This finding mirrors previous studies [37, 38] that identified significant mediation by mental health variables in the connection between adolescent psychological variables and life satisfaction. Therefore, to improve life satisfaction in late adolescents, it is crucial to implement strategies to improve mental health. These strategies may include mental health-related screening tests, proactive counseling, and the provision of systematic programs.

This study is noteworthy as it represents the first exploration utilizing extensive nationwide data from Korea to investigate the interplay between satisfaction with college education and life satisfaction in relation to career support, as well as the mediating effect of mental health in late adolescents. However, the significance of the relationship between the extent of career support assistance and mental health has not been elucidated, and related studies remain scarce. Consequently, it is imperative for future research to clarify the association between various career support programs and mental health improvement.

## Conclusion and implication

This study is of significance as it sheds light, for the first time to the best of the researcher’s knowledge, on the structural relationships among college education satisfaction, helpfulness of career assistance, and mental health in influencing the life satisfaction of Korean youth based on nationally collected data from the "2021 Youth Socio-Economic Reality Survey." The findings indicate that high satisfaction with college education not only has a direct positive impact on life satisfaction in Korean youth, but also serves as a significant factor indirectly affecting their life satisfaction by promoting mental health. The education sector has recently recognized the necessity for innovative educational curricula and methods that align with rapid societal changes, such as the Fourth Industrial Revolution. There is a growing emphasis on the need for qualitative improvement in college education. Therefore, colleges should regularly assess the satisfaction of students and, on the basis of the results of satisfaction surveys, actively incorporate the demands of education consumers into the curriculum. Furthermore, there is a need for efforts to move away from teacher-centered and standardized education toward effective education that focuses on enhancing students’ practical competencies through student-centered activities. It is also essential to be responsive to the demands of industries and society, incorporating these considerations into educational innovation at the university level. This should include providing educational programs that can be applied to future employment and the real-life scenarios faced by young people.

Additionally, this study revealed that as the perceived level of career support assistance increased, the life satisfaction of young people improved. Therefore, career support programs should be designed and delivered in a way that young people can tangibly experience their connection to career decision-making and employment. Furthermore, mental health not only has a highly significant direct impact on the life satisfaction of young people, but also acts as a mediating factor that reinforces the relationship between satisfaction with college education and overall life satisfaction. Therefore, special attention from relevant experts and practitioners in universities and local communities is necessary to improve the mental health of young individuals. It would be effective to identify mental health issues among young people and implement systematic, targeted, and tailored strategies, such as individualized programs, to promote mental health based on their specific mental health concerns.
